# LncRNA PRRT3-AS1 exerts oncogenic effects on nonsmall cell lung cancer by targeting microRNA-507/homeobox B5 axis

**DOI:** 10.32604/or.2022.026236

**Published:** 2022-11-10

**Authors:** RUI ZHOU, JIANYANG XU, LINGWEI WANG, JIANXIN LI

**Affiliations:** Department of Thoracic Surgery, Affiliated Zhongshan Hospital of Dalian University, Dalian, 116011, China

**Keywords:** Targeted therapy, lncRNA, microRNA, NSCLC

## Abstract

Long noncoding RNAs (lncRNAs) act as key regulators controlling complex cellular behaviors in nonsmall cell lung cancer (NSCLC). We investigated the expression of lncRNA PRRT3 antisense RNA 1 (PRRT3-AS1) in paired samples of NSCLC and adjacent normal tissues from a patient cohort in our hospital using real-time quantitative reverse transcription polymerase chain reaction (qRT-PCR) and found that it was significantly higher in NSCLC tissue than in normal tissue, consistent with The Cancer Genome Atlas database. Furthermore, functional investigation revealed that lncRNA PRRT3-AS1 depletion inhibited NSCLC-cell proliferation, colony formation, invasion, and migration, whereas its overexpression exerted the opposite effects. Moreover, PRRT3-AS1 knockdown suppressed *in vivo* NSCLC growth. Investigation of downstream mechanisms using RNA immunoprecipitation and luciferase reporter assay revealed that lncRNA PRRT3-AS1 acted as a competing endogenous RNA by adsorbing microRNA-507 (miR-507) and enhanced the expression of its target gene, homeobox B5 (HOXB5), in NSCLC. Furthermore, miR-507 downregulation or HOXB5 upregulation eliminated the cancer-inhibiting effects of lncRNA PRRT3-AS1 depletion in NSCLC cells. To conclude, the lncRNA PRRT3-AS1/miR-507/HOXB5 pathway acts as a promoter of malignant characteristics in NSCLC, and this newly identified competing endogenous RNA pathway may be an effective diagnostic, prognostic, and therapeutic target in NSCLC.

## Introduction

Among all cancers in humans, lung cancer has the highest incidence and mortality rate globally [1], with approximately 2.1 million lung cancer cases and over 1.8 million mortalities annually. Nonsmall cell lung cancer (NSCLC) is the predominant type of lung cancer, accounting for approximately 85% of all lung cancer cases [2]. Although advances in therapeutics have significantly improved the prognosis of NSCLC patients in recent decades, the therapeutic efficacy remains unsatisfactory, and many NSCLC patients still die [3]. The NSCLC pathogenesis and development is a complicated multistep process involving many risk factors and genes, and the underlying molecular mechanisms are poorly understood [4]. Therefore, determining the mechanisms underlying NSCLC pathogenesis is vital to identify effective targets for NSCLC diagnosis, prevention, and management.

Long noncoding RNAs (lncRNAs) are a family of RNA molecules comprising 200 or more nucleotides with no protein-coding ability [5,6]. In the past, lncRNAs were considered “transcriptional noise” or “dark matter” [7–9]. However, recent studies have shown that lncRNAs are crucial for regulating physiological and pathological processes, such as chromatin remodeling, cell differentiation, and carcinogenesis [10–13], as well as cancer progression [14–16]. Recently, several lncRNAs were reported to be dysregulated in NSCLC [17–19], with upregulated lncRNAs playing an oncogenic role to promote NSCLC progression, whereas weakly expressed lncRNAs playing an anticancer role [20–22].

MicroRNAs (miRNAs) are a family of short (approximately 17–23 nucleotides) endogenous noncoding RNA molecules [23]. MiRNA dysregulation, widely reported in NSCLC, affects malignant properties [24–26]. During the past decade, the theory of competing endogenous RNAs (ceRNAs) has been proposed [27], notably showing that lncRNAs can sequester miRNAs, thereby increasing the downstream targets of miRNAs [28]. Therefore, characterizing the functions of novel NSCLC-associated lncRNAs or miRNAs is critical for developing promising therapeutic targets.

Several lncRNAs are known to be key regulators of NSCLC malignancy. We investigated the expression and detailed functions of PRRT3 antisense RNA 1 (PRRT3-AS1) in NSCLC, in addition to the downstream mechanisms.

## Materials and Methods

### Clinical tissue samples

We obtained NSCLC tissues from 53 patients in our hospital who had not undergone chemotherapy or radiotherapy before surgery. Immediately after tissue excision, all tissue samples were immersed and stored in liquid nitrogen until further analysis. This research was approved by the ethics committee of the Affiliated Zhongshan Hospital of Dalian University. In addition, all participating patients provided written informed consent.

### Cell cultures

We purchased human NSCLC cell lines A549, H460, SK-MES-1, and H1299 from the American Type Culture Collection (ATCC; Manassas, VA, USA). Bronchial epithelial cell growth medium (Lonza; Walkersville, MD, USA) was adopted for culturing a human nontumorigenic bronchial epithelial cell line BEAS-2B (ATCC). Cell lines A549 and H1299 were grown in RPMI-1640 medium (Gibco). SK-MES-1 and H460 cell lines were grown in F-12K and MEM (Gibco), respectively. Each of the above culture media were supplemented with 10% fetal bovine serum (FBS) and 1% penicillin-streptomycin (Gibco). All cells were grown in a humidified atmosphere of 5% CO_2_ at 37°C.

### Transfection

We purchased small interfering RNAs (siRNAs) against PRRT3-AS1 (si-PRRT3-AS1) and homeobox B5 (HOXB5; si-HOXB5) and negative control (NC) siRNA (si-NC) from GenePharma (Shanghai, China). In addition, we used the miR-507 mimic and miR-507 inhibitor (RiboBio Co., Ltd. Guangzhou, China) to knock down and upregulate endogenous miR-507 levels, respectively. We used miRNA mimic (miR-NC) and inhibitor NCs as controls. The PRRT3-AS1 overexpression plasmid pcDNA3.1-PRRT3-AS1 (pc-PRRT3-AS1) and HOXB5 overexpression plasmid pcDNA3.1-HOXB5 (pc-HOXB5) were designed and synthesized by GenePharma. NSCLC cells were seeded into 6-well plates, and cell transfection was executed when cell growth reached 80% confluence. Cellular transfection was done by applying Lipofectamine® 2000 (Invitrogen, Carlsbad, CA, USA) and the vectors to the wells.

### Quantitative reverse transcription polymerase chain reaction

We used the TRIzol® reagent (Invitrogen) to extract total RNA. Total RNA was reverse-transcribed by using the Mir-X miRNA First-Strand Synthesis Kit (TaKaRa; Dalian, China). With the obtained cDNA as a template, we performed quantitative polymerase chain reaction (PCR; qPCR) to determine miR-507 expression by using the Mir-X miRNA quantitative reverse transcription PCR (qRT-PCR) TB Green® Kit (TaKaRa). U6 served as the endogenous control for miR-507. To measure PRRT3-AS1 and HOXB5 expression, we used the PrimeScript® RT Reagent Kit for cDNA synthesis and SYBR® Premix Ex Taq™ II (TaKaRa) for qPCR. PRRT3-AS1 and HOXB5 levels were normalized to GAPDH the internal control, and reported as log-fold changes using the 2^−ΔΔCq^ formula.

### Cell Counting Kit-8 and colony formation assays

We inoculated transfected NSCLC cells into 96-well plates with a density of 2000 cells per well and incubated them at 37°C in a 5% CO_2_ atmosphere. After incubating with 10 µL of the cell counting kit-8 (CCK-8) solution (Beyotime), we estimated the proliferation rate by the measurement of optical density at 450 nm. We performed the assay every day until day 4 and plotted the results as a growth curve.

For colonies, a cell suspension was prepared, and 2 ml of the cell suspension containing 600 cells was added into each well of 6-well plates. After 14 days of cultivation, the newly formed colonies were fixed with 100% methanol and stained with 0.1% crystal violet. The cells in the colonies were manually counted using an inverted microscope (x200 magnification).

### Transwell cell migration and invasion assays

We explored the invasive ability of NSCLC cells using Transwell inserts (BD Biosciences). We added 100 µL of Matrigel (BD Biosciences) into the inner sides of the Transwell inserts and performed polymerization by incubating them at 37°C for 2 h. Next, we loaded the upper chambers with 100 μL of FBS-free culture medium containing 4 × 10^4^ of transfected cells, and plated 600 μL of culture medium supplemented with 10% FBS into the lower chambers. We allowed the NSCLC cells to penetrate the pores in the submembrane surface for 24 h, then fixed them with 100% methanol, and stained them with 0.1% crystal violet. The cells that invaded the lower chambers were counted in five randomly chosen regions (in each well) under an inverted microscope. For the migration assay, we did not apply Matrigel, but the remaining steps were the same as those in the Matrigel invasion assay.

### Xenograft tumor assay

The short hairpin RNA (shRNA) targeting PRRT3-AS1 (sh-PRRT3-AS1), and NC shRNA (sh-NC) were designed and chemically generated by GenePharma. We inserted the shRNAs into a lentiviral vector to transfect the shRNAs into H460 cells. The PRRT3-AS1-knockdown H460 cells were selected by cultivating them with puromycin. Next, a total of 8 BALB/c nude mice (age, 4–6 weeks) were obtained from HFK Bioscience (Beijing, China) and randomly divided into sh-PRRT3-AS1 and sh-NC groups. A total of 2 × 10^6^ PRRT3-AS1-knockdown H460 cells were subcutaneously inoculated into each mouse in the sh-PRRT3-AS1 group. We monitored the width and length of tumor xenografts from 1 week after cell inoculation and then every 5 days. Thirty-two days later, all mice were anesthetized by means of cervical dislocation, and the tumor xenografts were completely detached and weighed.

All animal experiments were approved by the Animal Care and Use Committee of Affiliated Zhongshan Hospital of Dalian University. The humane endpoints were tumor diameter >1.5 cm, tumor ulceration, abnormal feeding, weight loss, ascites and cachexia. Meanwhile, no anesthesia was applied in the animal experiments.

### Fluorescence in situ hybridization

The subcellular location of PRRT3-AS1 in NSCLC cells was examined using the Fluorescent *In Situ* Hybridization (FISH) Kit (RiboBio Co., Ltd.,). In detail, after washing with phosphate-buffered saline, H460 and A549 cells were fixed with 4% paraformaldehyde, followed by the hybridization treatment specifically targeting PRRT3-AS1 (RiboBio Co., Ltd.) at 37°C without light. On the next day, DNA staining was performed using Hoechst solution. The cells were, imaged under a confocal laser scanning microscope (Leica; Solms, Germany).

### Bioinformatics prediction

We searched the miRNA Target Prediction DataBase (miRDB) (http://mirdb.org/) database for PRRT3-AS1’s target miRNAs. To predict the downstream target of miR-507, we used Targetscan (https://www.targetscan.org/vert_80/) applied to miRDB.

### RNA immunoprecipitation assay

We performed a RNA immunoprecipitation (RIP) assay using the Magna RIP RNA-Binding Protein Immunoprecipitation kit (Merck-Millipore; Bedford, MA, USA). Briefly, we harvested 80% confluent NSCLC cells and lysed them using the RIP lysis buffer. Next, we performed Argonaute 2 (Ago2) immunoprecipitation by incubating the cell lysate with magnetic beads conjugated with anti-Ago2 antibody or normal immunoglobulin G (IgG) (control; Merck Millipore). Ago2 is an important element of RNA-induced silencing complex, and it can promote the degradation of target mRNAs via its catalytic activity in gene silencing processes induced by miRNAs. After overnight incubation at 4°C, we extracted the immunoprecipitated RNA and evaluated the abundance of lncRNA PRRT3-AS1, miR-507, and HOXB5 mRNA using qRT-PCR.

### Luciferase reporter assay

The PRRT3-AS1 and HOXB5 wild-type (WT) sequences, both enclosing the predicted miR-507-binding sequences, were amplified by GenePharma, and fused to the psiCHECK™-2 luciferase reporter vector (Promega), generating the WT-PRRT3-AS1 and WT-HOXB5 reporter vectors. Similarly, the mutant (MUT) luciferase reporter vectors MUT-PRRT3-AS1 and MUT-HOXB5 were built by inserting PRRT3-AS1 and HOXB5 mutant sequences into the psiCHECK™-2 vector. Next, NSCLC cells were transfected with WT or matched MUT reporter vectors alongside the miR-507 mimic or miR-NC. On post-transfection day 2, we detected luciferase activity by applying the dual-luciferase reporter assay (Promega).

### Western blotting

We performed total protein extraction and quantification using the Pierce RIPA lysis buffer and the Pierce™ bicinchoninic acid (BCA) Kit (both from Thermo Fisher Scientific; MA, USA), respectively. Protein samples were separated by 10% SDS-PAGE and blotted onto PVDF membranes. After blocking them with 5% defatted milk powder, the membranes were incubated at 4°C overnight with the primary antibodies against Hoxb5 (ab109375) or Gapdh (ab128915), followed by incubation with the HRP-conjugated anti-rabbit secondary antibody (ab6721; all from Abcam, USA). Finally, the bands of target proteins were developed by treatment with an enhanced chemiluminescence reagent (Beyotime).

### Statistical analysis

All experiments were independently repeated three times. Results are reported as the mean ± 1 SD. The between-group differences were compared using Student’s *t*-test or ANOVA. *p* < 0.05 was considered statistically significant.

## Results

### PRRT3-AS1 promotes the aggressive behaviors of NSCLC cells

**Figure 1:**
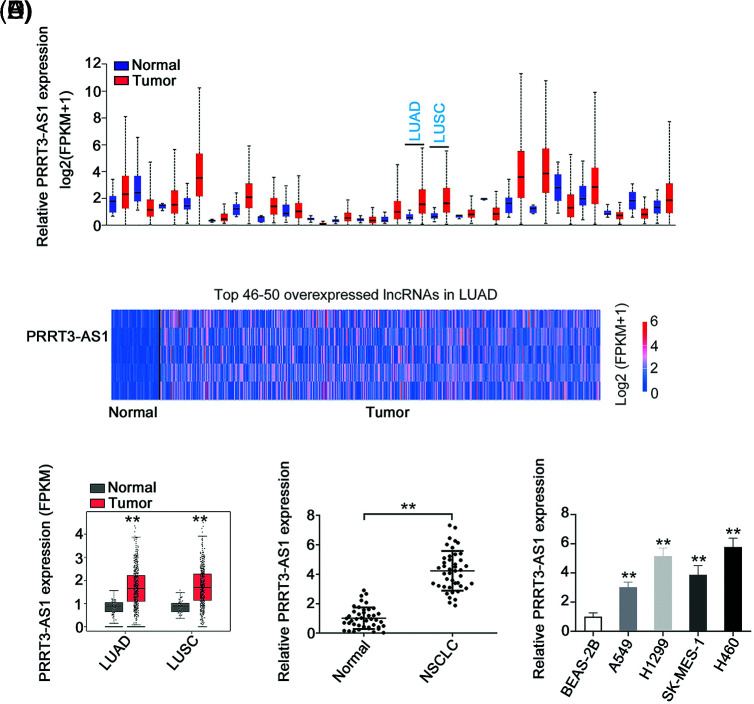
PRRT3-AS1 is upregulated in NSCLC. (A) PRRT3-AS1 level in all human cancer types from TCGA dataset (N = 3). (B) PRRT3-AS1 ranks the 47^th^ overexpressed lncRNA in LUAD. (C) PRRT3-AS1 level in LUAD and LUSC tissues from the TCGA database. (D) PRRT3-AS1 level in NSCLC tissues from our cohort (N = 3). (E) PRRT3-AS1 level in NSCLC cell lines (N = 3). **p < 0.001.

PRRT3-AS1 expression in human cancers was first investigated by analyzing The Cancer Genome Atlas dataset. We found that this gene was upregulated in nearly all human cancer types (Fig. 1A). Additionally, PRRT3-AS1 was the 47^th^ overexpressed lncRNA in lung adenocarcinoma (LUAD; Fig. 1B). Also, relative to control samples, PRRT3-AS1 was obviously overexpressed in lung squamous cell carcinoma and LUAD samples in the TCGA database (Fig. 1C). Next, we collected paired samples of NSCLC and adjacent normal tissues from 53 patients and determined the lncRNA PRRT3-AS1 levels in both sets. The qRT-PCR analysis revealed that PRRT3-AS1 was strongly expressed in NSCLC tissues (Fig. 1D). In addition, all four NSCLC cell lines manifested relatively higher lncRNA PRRT3-AS1 levels than the BEAS-2B (non-tumor control) cells (Fig. 1E).

**Figure 2:**
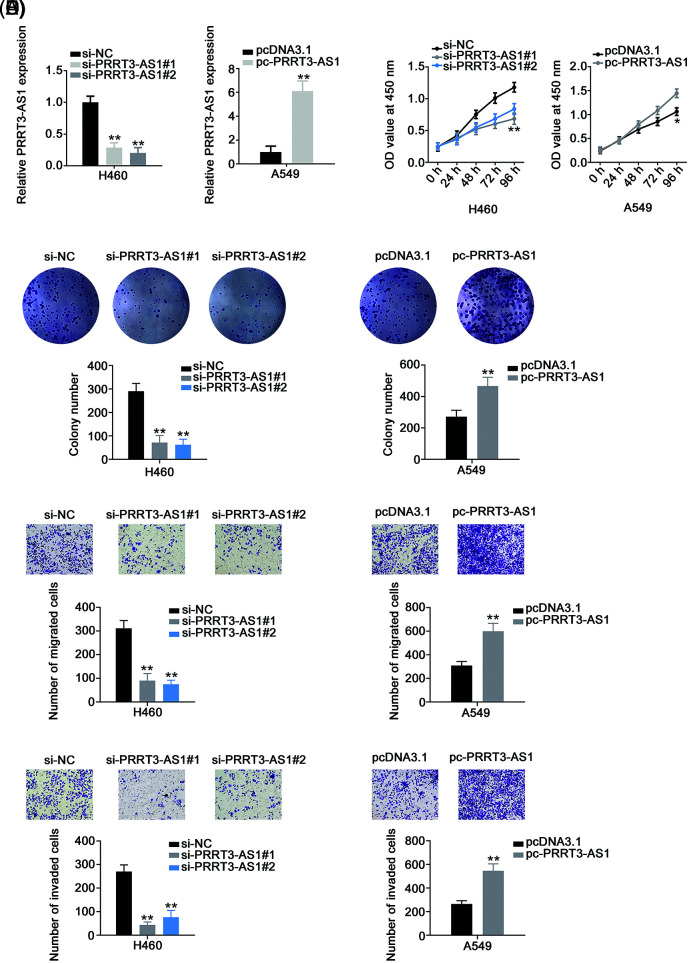
PRRT3-AS1 facilitates the malignant properties of NSCLC cells. (A) Inhibition efficiency of two siRNAs targeting PRRT3-AS1 in H460 cells. The efficiency of pc-PRRT3-AS1 was also explored in A549 cells (N = 3). (B) Cell proliferation of PRRT3-AS1-knockdown H460 cells and PRRT3-AS1-overexpressed A549 cells (N = 3). (C) The colony formation of si-PRRT3-AS1-transfected H460 cells and pc-PRRT3-AS1-transfected A549 cells (N = 3). (D) The migration of si-PRRT3-AS1-transfected H460 cells and pc-PRRT3-AS1-transfected A549 cells (N = 3) 100× magnification. (E) The invasion of si-PRRT3-AS1-transfected H460 cells and pc-PRRT3-AS1-transfected A549 cells (N = 3) 100× magnification. *p < 0.01 and **p < 0.001.

We knocked down PRRT3-AS1 in H460 cells by transfecting them with si-PRRT3-AS1. To avoid the off-target effect, we used two siRNAs and verified that both manifested effective PRRT3-AS1 knockdown. Meanwhile, we applied pc-PRRT3-AS1 to overexpress PRRT3-AS1 in A549 cells (Fig. 2A), and then examined the biological effects of PRRT3-AS1 in the cells. PRRT3-AS1 knockdown clearly inhibited the proliferation of H460 cells, whereas PRRT3-AS1 overexpression promoted A549 cell proliferation (Fig. 2B). In addition, colony formation was clearly restricted in H460 cells following PRRT3-AS1 ablation, while PRRT3-AS1 upregulation caused the opposite tendency in A549 cells (Fig. 2C). Furthermore, PRRT3-AS1 knockdown decreased the motility (Figs. 2D and 2E) of H460 cells, whereas transfection with pc-PRRT3-AS1 resulted in the enhancement of A549 cell motility (Figs. 2D and 2E). Together, these results indicate that lncRNA PRRT3-AS1 performed oncogenic actions during NSCLC progression.

### PRRT3-AS1 acts as a miR-507 sponge in NSCLC

**Figure 3:**
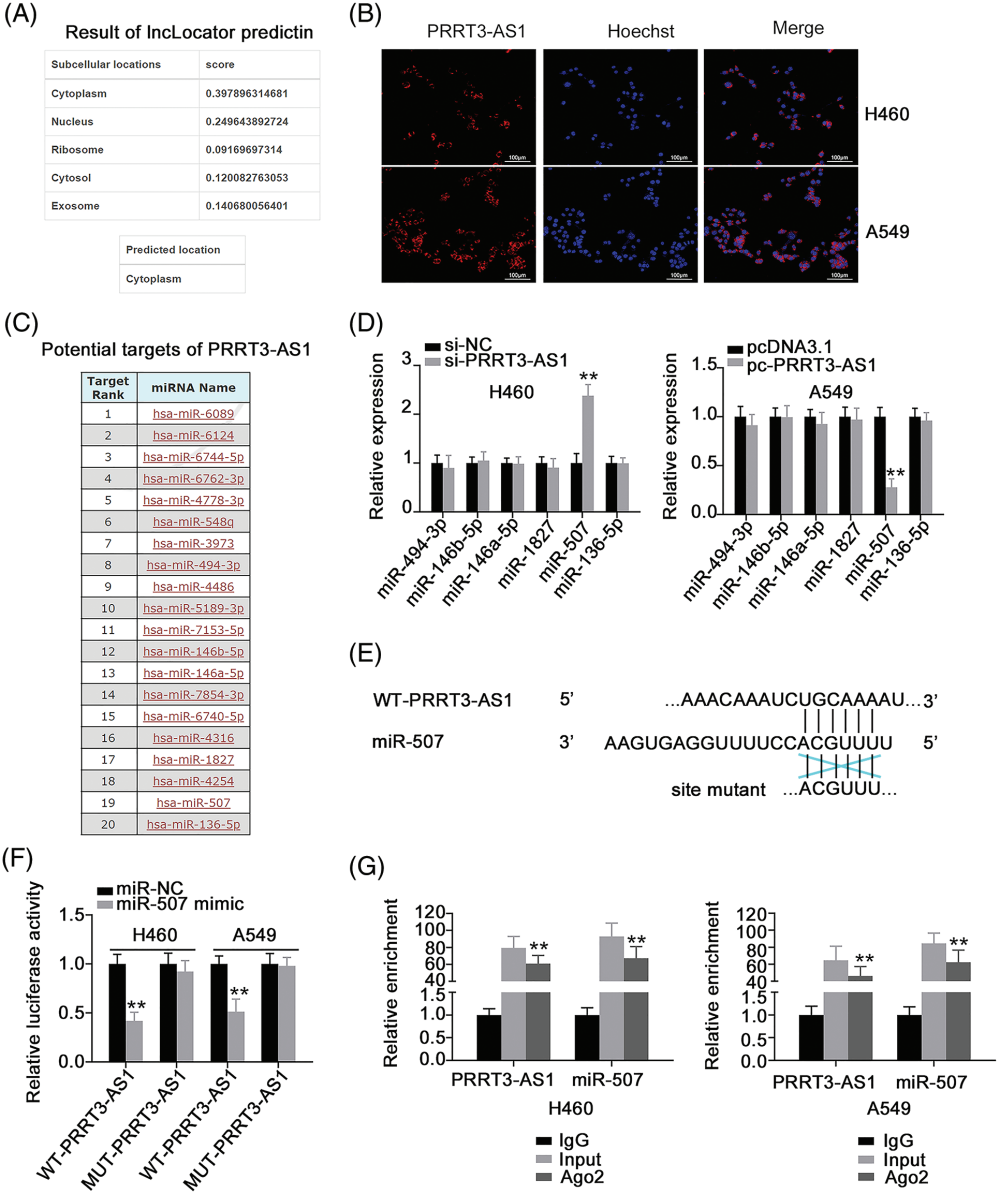
PRRT3-AS1 is an miR-507 sponge in NSCLC cells. (A) Predicted location of PRRT3-AS1 according to lncLocator. (B) Subcellular distribution of PRRT3-AS1 in NSCLC cells. (C) miRDB online database to predict potential miRNA-targeting PRRT3-AS1. (D) Levels of the six candidates in H460 cells after PRRT3-AS1 depletion. Also, the levels of six candidates in A549 cells after PRRT3-AS1 upregulation was also investigated (N = 3). (E) The WT and MUT binding sites of miR-507 within PRRT3-AS1. (F) Luciferase reporter assay implemented in NSCLC cells after cotransfection with the miR-507 mimic or miR-NC and WT-PRRT3-AS1 or MUT-PRRT3-AS1 (N = 3). (G) RIP assay to analyze the interaction between PRRT3-AS1 and miR-507 (N = 3). **p < 0.001.

To determine downstream mechanisms of PRRT3-AS1 activation, we detected the subcellular localization of PRRT3-AS1 in NSCLC cells. First, to predict lncRNA PRRT3-AS1’s subcellular location, we used lncLocator (http://www.csbio.sjtu.edu.cn/bioinf/lncLocator/), which indicated that it was mostly located in the cytoplasm (Fig. 3A). We verified the prediction by using FISH, confirming that PRRT3-AS1 was indeed a cytoplasmic lncRNA (Fig. 3B). This observation suggested that lncRNA PRRT3-AS1 may act as a ceRNA of miRNAs. To follow up on this lead, we performed a bioinformatic analysis of the miRDB database revealing that lncRNA PRRT3-AS1 had potential binding sites for 20 miRNAs (Fig. 3C). A search of the literature identified six miRNAs, including miR-494-3p [29], miR-146b-5p [30], miR-146a-5p [31], miR-1827 [32], miR-507 [33], and miR-136-5p [34], which were shown to be significantly correlated with NSCLC progression. Therefore, we selected these six miRNAs for further analysis.

We investigated the regulatory effects of lncRNA PRRT3-AS1 on the six miRNAs and found that miR-507 was the one that changed the most (Fig. 3D). Luciferase reporter assay was adopted to confirm the binding between lncRNA PRRT3-AS1 and miR-507 (Fig. 3E) and indicated that upregulated miR-507 expression decreased the luciferase activity of WT-PRRT3-AS1, while MUT-PRRT3-AS1 activity remained unaffected after miR-507 upregulation (Fig. 3F). Additionally, PRRT3-AS1 and miR-507 were abundant in the RNA immunoprecipitated by anti-Ago2 antibody (Fig. 3G). Overall, these results indicate that lncRNA PRRT3-AS1 functioned as a miR-507 sponge.

### HOXB5 is directly targeted by miR-507 in NSCLC cells and controlled by the lncRNA PRRT3-AS1/miR-507 axis

**Figure 4:**
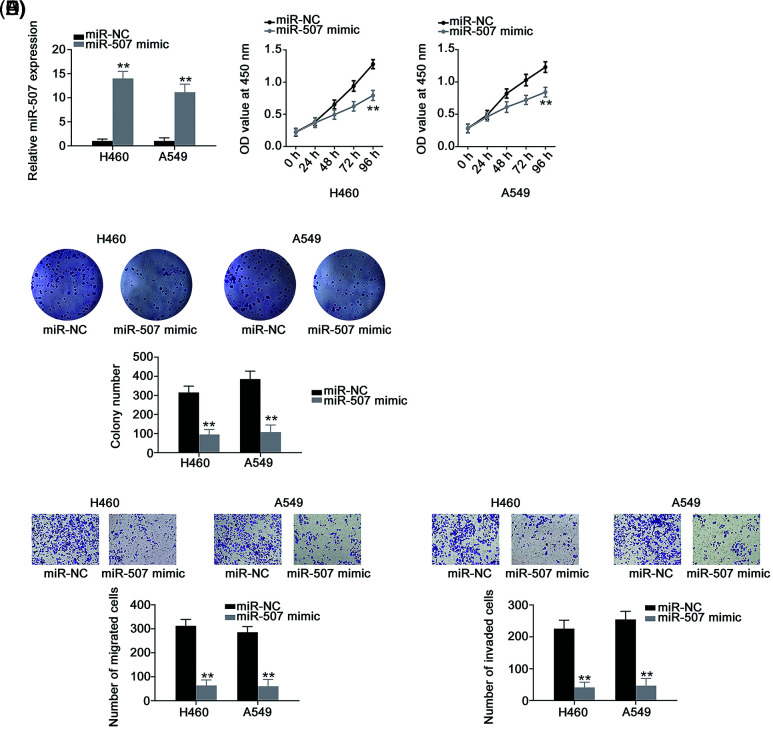
Overexpressed miR-507 inhibits the malignant process of NSCLC cells. (A) The efficiency of miR-507 mimic transfection in NSCLC cells (N = 3). (B, C) Cell proliferation and colony formation of miR-507-overxressed NSCLC cells (N = 3). (D, E) The motility of miR-507-overexpressed NSCLC cells (N = 3) 100× magnification. **p < 0.001.

**Figure 5:**
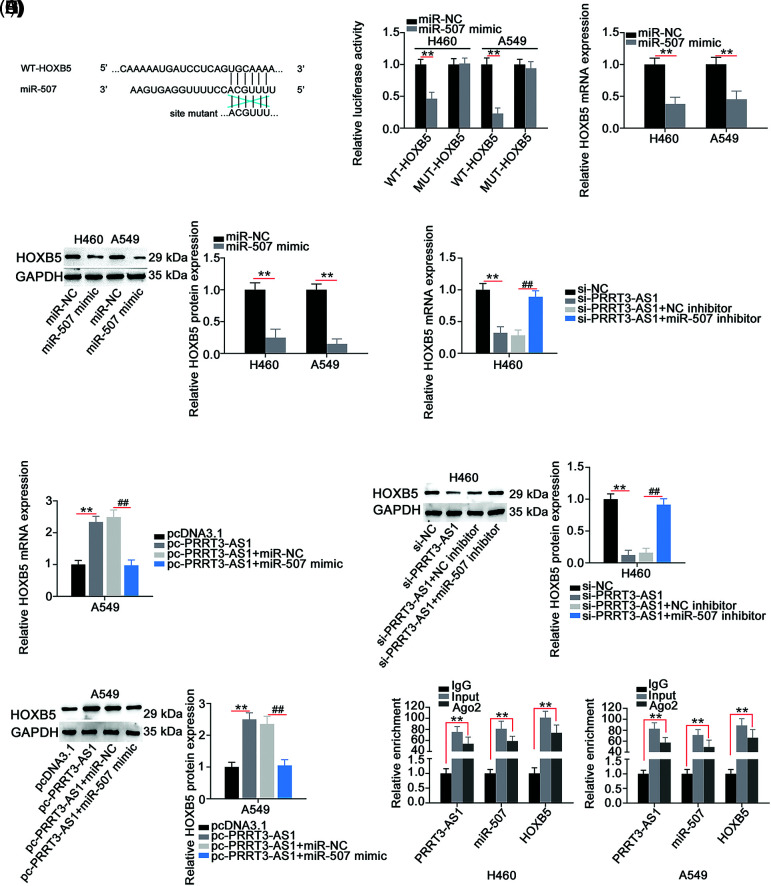
HOXB5 is under the control of PRRT3-AS1/miR-507 axis. (A) The WT and MUT binding sites between miR-507 and HOXB5 3′-UTR. (B) Luciferase reporter assay to verify binding interaction between miR-507 and HOXB5 3′-UTR in NSCLC cells (N = 3). (C and D) HOXB5 levels in miR-507-overxressed NSCLC cells (N = 3). (E–H) PRRT3-AS1-silenced H460 cells were cotransfected with miR-507 inhibitor or NC inhibitor, while miR-507 mimic or miR-NC was transfected into PRRT3-AS1-overexrssed A549 cells. After transfection, HOXB5 mRNA and protein levels were examined (N = 3). (I) RIP assay to certify the interaction among PRRT3-AS1, miR-507, and HOXB5 (N = 3). **p < 0.001 and ^##^*p* < 0.001.

Next, we determined the roles of miR-507 overexpression (Fig. 4A) in NSCLC cells. We found that miR-507 enhanced tumor-suppressing activities in NSCLC cells, affecting multiple aggressive behaviors (Figs. 4B–4E). Since HOXB5 (Fig. 5A) was predicted as a potential target of miR-507, we next analyzed whether miR-507 directly targeted HOXB5 in NSCLC cells. We found that miR-507 overexpression decreased the luciferase activity of the WT-HOXB5 vector in NSCLC cells, whereas no change was observed for the MUT-HOXB5 luciferase vector (Fig. 5B). In addition, our data showed a significant decrease in HOXB5 levels (Figs. 5C and 5D) in miR-507-overexpressed-NSCLC cells. All these results together demonstrate that HOXB5 is a downstream target of miR-507 in NSCLC cells.

Given that lncRNA PRRT3-AS1 sequestered miR-507 in NSCLC, we hypothesized that lncRNA PRRT3-AS1 indirectly modulated HOXB5 expression through sequestering miR-507. In fact, HOXB5 levels were decreased in H460 cells after lncRNA PRRT3-AS1 depletion, whereas HOXB5 levels were increased in pc-PRRT3-AS1-transfected A549 cells (Figs. 5E–5H). In addition, si-PRRT3-AS1-induced inhibition of HOXB5 expression in H460 cells recovered after cotransfection with an miR-507 inhibitor, while treatment with a miR-507 mimic lead to recovery from the increased HOXB5 expression induced by pc-PRRT3-AS1 in A549 cells (Figs. 5E–5H). The RIP assay validated that lncRNA PRRT3-AS1, miR-507, and HOXB5 were abundant in products immunoprecipitated by the anti-Ago2 antibody (Fig. 5I). Together, these results showed that lncRNA PRRT3-AS1 decoyed miR-507 in NSCLC cells and thus, positively modulate HOXB5 expression.

### LncRNA PRRT3-AS1 worsens the oncogenicity of NSCLC cells via targeting the miR-507/HOXB5 axis

**Figure 6:**
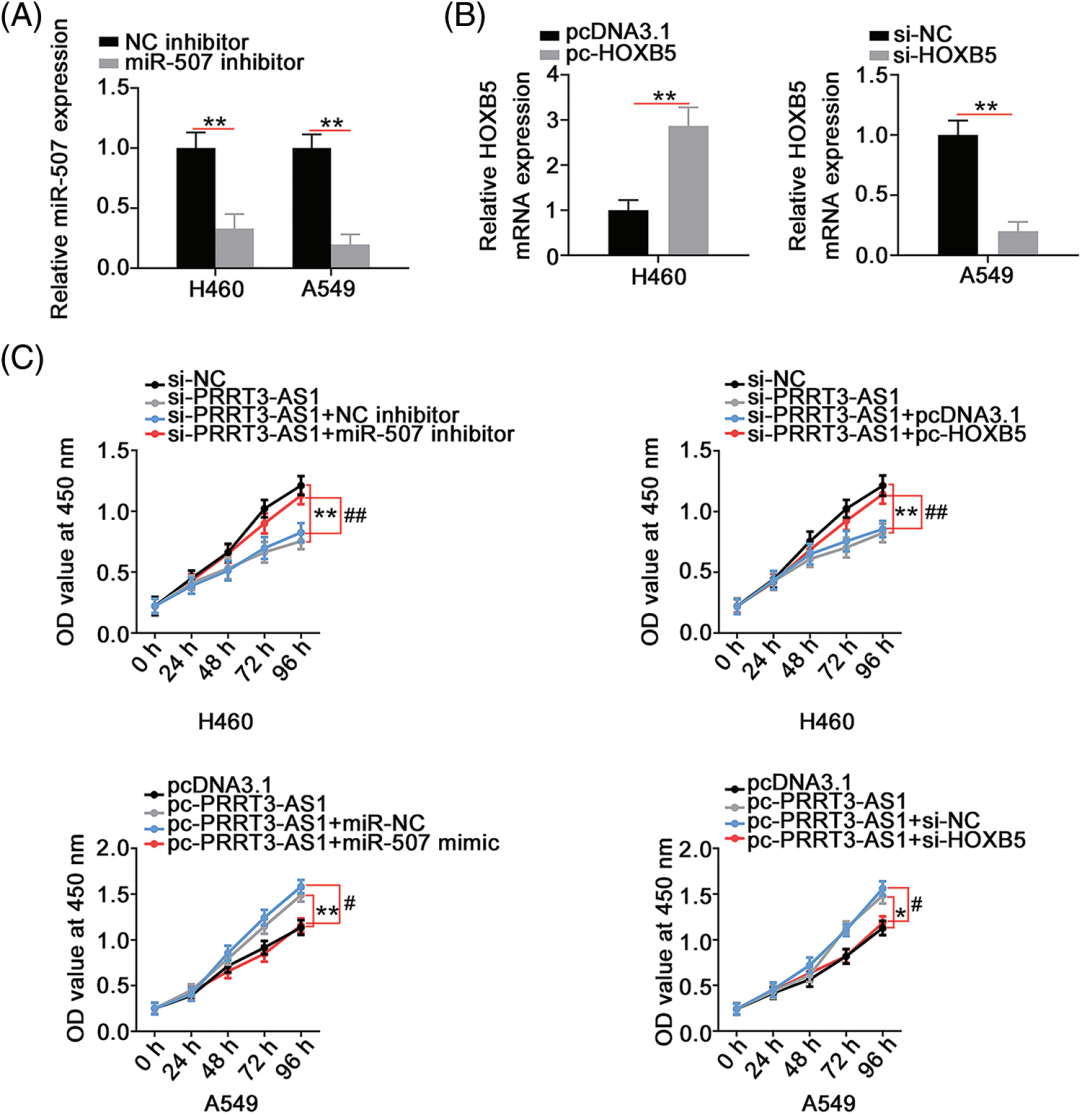
PRRT3-AS1 regulates NSCLC cell proliferation via targeting miR-507/HOXB5. (A) Inhibition efficiency of miR-507 inhibitor in NSCLC cells (N = 3). (B) The efficiency of pc-HOXB5 in H460 cells and si-HOXB5 in A549 cells (N = 3). (C) PRRT3-AS1-silenced H460 cells were cotransfected with miR-507 inhibitor or pc-HOXB5 while miR-507 mimic or si-HOXB5 was introduced into PRRT3-AS1-overexrssed A549 cells. After transfection, cell proliferation was determined via CCK-8 assay (N = 3). *p < 0.01, **p < 0.001, ^#^*p* < 0.01, and ^##^*p* < 0.001.

We designed rescue experiments to determine whether lncRNA PRRT3-AS1 performed tumor-promoting actions in NSCLC cells by controlling the miR-507/HOXB5 axis. First, we tested the transfection efficiency of the miR-507 inhibitor in NSCLC cells, verifying that miR-507 significantly decreased in miR-507 inhibitor-transfected cells (Fig. 6A). Meanwhile, the efficiency of pc-HOXB5 in H460 cells and si-HOXB5 in A549 cells was explored (Fig. 6B).

**Figure 7:**
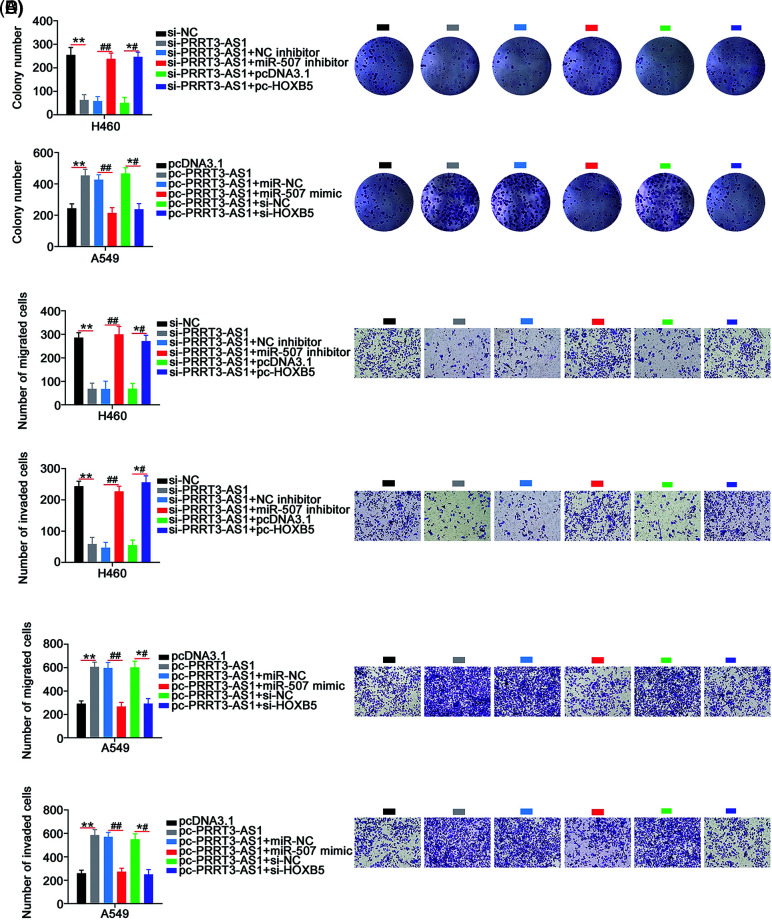
PRRT3-AS1 promotes NSCLC cell colony formation and motility through controlling miR-507/HOXB5. (A) The si-PRRT3-AS1-transfected H460 cells underwent cotransfection with miR-507 inhibitor or pc-HOXB5. Cell colony formation was then examined (N = 3). (B) miR-507 mimic or si-HOXB5 alongside pc-PRRT3-AS1 was cotransfected into A549 cells, and then subjected to colony formation determination (N = 3). (C) The si-PRRT3-AS1 together with miR-507 inhibitor or pc-HOXB5 was transfected into H460 cells. Cell motility was detected (N = 3) 100× magnification. (D) A549 cells were transfected with pc-PRRT3-AS1 alongside miR-507 mimic or si-HOXB5. Cell motility was also measured (N = 3) 100× magnification. **p < 0.001, ^##^*p* < 0.001 and *^#^*p* < 0.001.

Next, si-PRRT3-AS1 was combined with the miR-507 inhibitor or the pc-HOXB5 to transfect H460 cells. PRRT3-AS1-overexpresed A549 cells underwent cotransfection with the miR-507 mimic or si-HOXB5. The cell proliferation inhibited by si-PRRT3-AS1 was reversed by miR-507 inhibition or HOXB5 upregulation (Fig. 6C). Conversely, miR-507 overexpression or HOXB5 depletion recovered the increased cell proliferation in A549 cells transfected with pc-PRRT3-AS1 (Fig. 6C). Remarkably, interference with lncRNA PRRT3-AS1 hampered colony formation of H460 cells; conversely, inhibition of miR-507 or upregulation of HOXB5 prevented this influence (Fig. 7A). In summary, treatment with the miR-507 mimic or si-HOXB5 was capable of recovering the colony formation of A549 cells that was promoted by pc-PRRT3-AS1 (Fig. 7B).

In addition, the introduction of the miR-507 inhibitor or pc-HOXB5 offset the impaired H460 cell migration and invasion (Fig. 7C) induced by PRRT3-AS1 downregulation. Moreover, the cell migration and invasion abilities of cells transfected with pc-PRRT3-AS1 significantly increased, while those of cells transfected with miR-507 mimic or si-HOXB5 decreased this effect (Fig. 7D). To conclude, lncRNA PRRT3-AS1 worsened the oncogenicity of NSCLC, at least, in part, by targeting the miR-507/HOXB5 regulatory axis.

### PRRT3-AS1 depletion impairs growth of NSCLC cells in vivo

**Figure 8:**
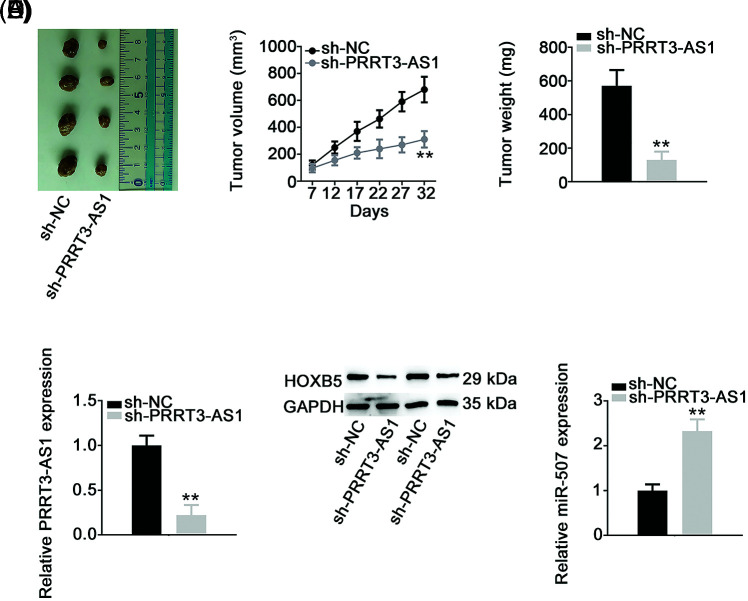
PRRT3-AS1 depletion decreases tumor growth *in vivo*. (A) Representative photographs of harvested tumors. Each group contained four nude mice. (B) Growth curve of tumor xenografts. (C) The comparison of the weight in the tumor xenografts. (D, E) PRRT3-AS1 and HOXB5 expression in tumor xenografts. (F) miR-507 level in tumor xenografts. **p < 0.001.

Xenograft tumor assay was performed to illustrate whether lncRNA PRRT3-AS1 affected NSCLC tumor growth *in vivo*. In contrast to tumors in the sh-NC group, the sh-PRRT3-AS1-transfected subcutaneous tumors grew significantly slower (Figs. 8A and 8B). Also, tumor weight was much lower in the sh-PRRT3-AS1 group in comparison with that in the sh-NC group (Fig. 8C). Furthermore, lncRNA PRRT3-AS1 (Fig. 8D) and HOXB5 (Fig. 8E) levels were reduced in tumor xenografts obtained from the sh-PRRT3-AS1 group, and lncRNA PRRT3-AS1-depleted tumor xenografts manifested clearly higher miR-507 levels (Fig. 8F). Taken together, these results indicated that downregulation of PRRT3-AS1 hampered NSCLC tumor growth *in vivo*.

## Discussion

Many lncRNAs have been considered as crucial promoters or inhibitors of NSCLC progression [25], but more recently, several studies have highlighted other important roles played by lncRNAs in regulating complex cellular behaviors in NSCLC [35–37]. Therefore, finding novel cancer-associated lncRNAs and exploring their regulatory actions in NSCLC is necessary to identify promising targets for NSCLC treatment. To date, 33,829 lncRNAs have been verified in the human genome according to the ENCODE database [38]; however, for most of these, the biological role and working mechanisms are still unknown. Our aim in this study was to investigate the specific functions of the lncRNA PRRT3-AS1 in NSCLC and the underlying downstream mechanisms.

PRRT3-AS1 is overexpressed in prostate cancer, and the knockdown of PRRT3-AS1 suppresses cell viability, migration, and invasion, while promoting cell apoptosis [39]. However, the expression pattern, clinical implications, and functions of PRRT3-AS1 in NSCLC are still unknown. Our RNA expression analysis results revealed that PRRT3-AS1 was strongly expressed in NSCLC tissues, consistent with the information about PRRT3-AS1 in the TCGA database. Loss- and gain-of-function assays to determine comprehensively the biological functions of lncRNA PRRT3-AS1 in NSCLC progression showed that it played a pro-oncogenic role in NSCLC; PRRT3-AS1 knockdown evidently inhibited NSCLC cell growth and motility *in vitro*, whereas PRRT3-AS1 overexpression exerted the opposite effects. These results suggest that PRRT3-AS1 could be considered as a possible target for NSCLC diagnosis, prognosis, and management.

Next, we determined the mechanisms underlying the effect of lncRNA PRRT3-AS1 on malignant properties. The extensive research on the potential mechanisms of lncRNA function has mostly been dependent on the cellular localization of lncRNAs. Nuclear lncRNAs directly bind to proteins and regulate gene expression at the transcriptional level [40]. In contrast, one of the critical roles of lncRNAs distributed in the cell cytoplasm is to partake, as ceRNAs, in the post-transcriptional regulation of gene expression [41]. According to the ceRNA theory, lncRNAs adsorb specific miRNAs, disabling the interaction of miRNAs with their target mRNAs [41]. Using two different methods, lncLocator prediction and fluorescence *in situ* hybridization, we determined that the subcellular localization of lncRNA PRRT3-AS1 was primarily in the cytoplasm of NSCLC cells, offering a theoretical basis for lncRNA PRRT3-AS1 functions as a ceRNA.

Bioinformatic analysis of downstream targets of lncRNA PRRT3-AS1 predicted miR-507 as a possible lncRNA PRRT3-AS1-sequestered miRNA. Subsequent luciferase reporter and RIP assays confirmed that lncRNA PRRT3-AS1 acted as a miR-507 sponge in NSCLC cells. Mechanistic investigation also identified HOXB5 as a direct target of miR-507 in NSCLC cells, showing that lncRNA PRRT3-AS1 indirectly modulated HOXB5 in NSCLC cells by sponging miR-507. Therefore, we hypothesize that PRRT3-AS1, miR-507, and HOXB5 coexist in the same RNA-induced silencing complex. Overall, lncRNA PRRT3-AS1 acts as a ceRNA to directly interact with miR-507 and indirectly regulate HOXB5 expression in NSCLC cells, thereby forming the novel lncRNA PRRT3-AS1/miR-507/HOXB5 ceRNA pathway.

We have known that miR-507 is differentially expressed in several human cancers, including NSCLC [42]. Here, we report that miR-507 is downregulated in NSCLC. Our functional experiments further unveiled that miR-507 inhibited NSCLC cell proliferation, migration, and invasion. Mechanistically, HOXB5, a member of the HOX gene family, is a direct target of miR-507 in NSCLC. HOXB5 plays a carcinogenic role by regulating growth, metastasis, epithelial–mesenchymal transition, and tumorigenesis [43]. Our data supports the notion that HOXB5 is negatively regulated by miR-507 and positively modulated by lncRNA PRRT3-AS1 in NSCLC. In fact, our rescue experiments proved that miR-507 downregulation or HOXB5 upregulation eliminated the cancer-inhibiting effects of si-PRRT3-AS1. Together, our results strongly suggest that the PRRT3-AS1/miR-507/HOXB5 pathway functions as a promoter of malignant characteristics in NSCLC.

Our study had two limitations. Firstly, we did not explore the effect of PRRT3-AS1 on metastasis *in vivo*. Secondly, other ways may be involved in the mechanisms underlying the oncogenic roles of PRRT3-AS1 in NSCLC. We will resolve the two limitations in the near future.

To conclude, PRRT3-AS1, which is overexpressed in NSCLC, upregulates HOXB5 expression by sequestering miR-507 and aggravates the oncogenicity of NSCLC. Our findings advance the understanding of NSCLC pathogenesis and strongly suggest the PRRT3-AS1/miR-507/HOXB5 pathway as a promising novel therapeutic target.

## Data Availability

Data available with the communication author and can be provided upon reasonable request.
